# The causal effects of serum lipids and apolipoproteins on kidney function: multivariable and bidirectional Mendelian-randomization analyses

**DOI:** 10.1093/ije/dyab014

**Published:** 2021-06-21

**Authors:** Humaira Rasheed, Jie Zheng, Jessica Rees, Eleanor Sanderson, Laurent Thomas, Tom G Richardson, Si Fang, Ole-Jørgen Bekkevold, Endre Bakken Stovner, Maiken Elvestad Gabrielsen, Anne Heidi Skogholt, Solfrid Romundstad, Ben Brumpton, Stein Hallan, Cristen Willer, Stephen Burgess, Kristian Hveem, George Davey Smith, Tom R Gaunt, Bjørn Olav Åsvold

**Affiliations:** 1 K.G. Jebsen Center for Genetic Epidemiology, Department of Public Health and Nursing, NTNU, Norwegian University of Science and Technology, Trondheim, Norway; 2 MRC Integrative Epidemiology Unit, Population Health Sciences, Bristol Medical School, University of Bristol, Bristol, UK; 3 Department of Chemistry, University of Engineering and Technology, Lahore, Pakistan; 4 Cardiovascular Epidemiology Unit, University of Cambridge, Cambridge, UK; 5 Department of Clinical and Molecular Medicine, NTNU, Norwegian University of Science and Technology, Trondheim, Norway; 6 Department of Internal Medicine, Levanger Hospital, Nord-Trøndelag Hospital Trust, Levanger, Norway; 7 Department of Thoracic Medicine, St Olavs Hospital, Trondheim University Hospital, Trondheim, Norway; 8 Department of Nephrology, St Olavs Hospital, Trondheim University Hospital, Trondheim, Norway; 9 Department of Internal Medicine, University of Michigan, Ann Arbor, MI, USA; 10 MRC Biostatistics Unit, University of Cambridge, Cambridge, UK; 11 NIHR Bristol Biomedical Research Centre, University of Bristol, Bristol, UK; 12 Department of Endocrinology, Clinic of Medicine, St Olavs Hospital, Trondheim University Hospital, Trondheim, Norway

**Keywords:** Lipids, eGFR, Mendelian randomization, urinary albumin-to-creatinine ratio, apolipoproteins, kidney

## Abstract

**Background:**

The causal nature of the observed associations between serum lipids and apolipoproteins and kidney function are unclear.

**Methods:**

Using two-sample and multivariable Mendelian randomization (MR), we examined the causal effects of serum lipids and apolipoproteins on kidney function, indicated by the glomerular-filtration rate estimated using creatinine (eGFRcrea) or cystatin C (eGFRcys) and the urinary albumin-to-creatinine ratio (UACR). We obtained lipid- and apolipoprotein-associated genetic variants from the Global Lipids Genetics Consortium (*n* = 331 368) and UK Biobank (*n* = 441 016), respectively, and kidney-function markers from the Trøndelag Health Study (HUNT; *n* = 69 736) and UK Biobank (*n* = 464 207). The reverse causal direction was examined using variants associated with kidney-function markers selected from recent genome-wide association studies.

**Results:**

There were no strong associations between genetically predicted lipid and apolipoprotein levels with kidney-function markers. Some, but inconsistent, evidence suggested a weak association of higher genetically predicted atherogenic lipid levels [indicated by low-density lipoprotein cholesterol (LDL-C), triglycerides and apolipoprotein B] with increased eGFR and UACR. For high-density lipoprotein cholesterol (HDL-C), results differed between eGFRcrea and eGFRcys, but neither analysis suggested substantial effects. We found no clear evidence of a reverse causal effect of eGFR on lipid or apolipoprotein traits, but higher UACR was associated with higher LDL-C, triglyceride and apolipoprotein B levels.

**Conclusion:**

Our MR estimates suggest that serum lipid and apolipoprotein levels do not cause substantial changes in kidney function. A possible weak effect of higher atherogenic lipids on increased eGFR and UACR warrants further investigation. Processes leading to higher UACR may lead to more atherogenic lipid levels.

**Key Messages**

- Our Mendelian-randomization (MR) analyses indicate that serum lipids and apolipoproteins do not have a substantial impact on kidney function and urinary albumin excretion.
- There was some, but inconsistent, evidence that higher atherogenic lipid levels may weakly increase both the glomerular-filtration rate and urinary albumin-to-creatinine ratio (UACR); these findings need further replication.
- MR analyses provided no consistent evidence that the estimated glomerular-filtration rate influences serum lipid or apolipoprotein levels, but processes leading to higher UACR may increase the levels of atherogenic lipids including serum low-density lipoprotein cholesterol, triglycerides and apolipoprotein B.
- Our study was performed using data from general-population cohorts in which most participants had normal kidney function, so our results reflect associations within the healthy range of kidney function.

## Introduction

The increasing prevalence of chronic kidney disease (CKD) has become an important public-health concern.^1^ CKD causes 5–10 million deaths annually,^2^ including excess deaths from cardiovascular disease (CVD).^3^ The glomerular-filtration rate (GFR) estimated using serum creatinine (eGFRcrea) and albuminuria classified using the urinary albumin-to-creatinine ratio (UACR) are the most commonly used CKD-classifying quantitative traits.^4^^,^^5^ The GFR estimated using cystatin C (eGFRcys) may be an even better marker of kidney function than eGFRcrea, but is used less often clinically.^6^

Quantitative markers of kidney function are associated with dyslipidemia in which elevated triglyceride (TG) levels, decreased high-density lipoprotein cholesterol (HDL-C) and an increased TG-to-HDL-C ratio have been associated with lower eGFRcrea.^7^^,^^8^ Increased low-density lipoprotein cholesterol (LDL-C) and increased ratios of LDL-C to HDL-C and apolipoprotein B (Apo B) to A-I (Apo A-I) are associated with a decline in GFRcrea.^9^^,^^10^ It has been suggested that eGFRcys may be even more strongly correlated with unfavourable lipids than eGFRcrea.^11^ Further, elevated TG, Apo B and non-HDL-C levels have been associated with increased UACR in Asian cohorts.^12–14^ The causal role of these lipid and apolipoprotein traits in influencing kidney function (or vice versa) is unclear.

Mendelian randomization (MR) is an approach that can be used to estimate the causal associations between serum lipids and kidney-function markers, whilst minimizing the impact of reverse causality and confounding.^15^ Application of this approach in a recent study using 183 lipid-associated variants from the Global Lipids Genetics Consortium (GLGC)^16^ and associations of these same variants with eGFRcrea and UACR from a trans-ancestry meta-analysis genome-wide association study (GWAS)^17^ estimated that each 1-standard-deviation (SD) genetically predicted higher serum HDL-C caused 0.8% higher eGFRcrea and 4.4% lower UACR, with relatively similar causal estimates for LDL-C (0.5% and 3.8%) and TG (0.5% and 4.7%).^18^ Subsequently to this study, new genetic instruments for lipids have been discovered (444 genetic instruments based on 331 368 samples^19^). The lack of any beneficial effect of the HDL-C-raising drug niacin on kidney function in a recent clinical trial^20^ challenges the previously reported causal association of HDL-C. Further, there is a lack of MR studies examining whether apolipoproteins may influence kidney function or whether variation in kidney function influences lipid and apolipoprotein levels. To obtain a clearer causal understanding of the relationship between serum lipids and apolipoproteins and kidney-function markers, we performed univariable, multivariable and bidirectional MR in population-based cohorts from the Trøndelag Health Study (HUNT) in Norway and the UK Biobank (UKBB).

## Methods

### Study populations

We included participants of European ancestry with available genotype and phenotype data from the HUNT and UKBB studies (Supplementary Figure 1, available as Supplementary data at *IJE* online). HUNT is a series of general health surveys of the adult population of Trøndelag county, Norway.^21^ This study includes participants from the HUNT2 (1995–1997) and HUNT3 (2006–2008) surveys. Phenotypes available in HUNT included eGFRcrea, HDL-C, TG and LDL-C (details in Supplementary Methods, available as Supplementary data at *IJE* online). In UKBB, a broader set of phenotypes was available, including eGFRcrea, eGFRcys, UACR, HDL-C, TG, LDL-C, Apo A-I and Apo B (details in Supplementary Methods, available as Supplementary data at *IJE* online).

### Instrument selection

Out of 444 genetic variants (Supplementary Table 1, available as Supplementary data at *IJE* online) that have shown association with at least one of the lipid traits (HDL-C, LDL-C and TG) at a GWAS significance level in analyses of 331 368 participants in the GLGC,^19^ 390 single-nucleotide polymorphisms (SNPs) were available in HUNT. Among the missing 54 SNPs, 11 were removed during the harmonization and 43 have not been genotyped or imputed into HUNT (Supplementary Table 2A, available as Supplementary data at *IJE* online) and we either could not find any proxy SNP (as it was a rare variant) or the effect sizes for possible proxy (in close-linkage disequilibrium; LD) SNPs were not included in the reference GWAS. Similarly, 390 variants were available in UKBB and the remaining 54 variants were either missing or were removed during harmonization (Supplementary Table 2B, available as Supplementary data at *IJE* online). Out of 390 variants, as individual instruments for each lipid trait, we selected 168 SNPs associated with LDL-C, 193 SNPs associated with HDL-C and 172 SNPs associated with TG (Supplementary Table 3, available as Supplementary data at *IJE* online) at a GWAS significance level (*P*-value <5 × 10^–8^). This lipid GWAS used an LD threshold of *r*^2^ < 0.2, which is considered quite liberal for selecting independent variants. Thus, to further confirm our findings, we conducted sensitivity analyses applying a threshold of *r*^2^ < 0.001. The genetic variants associated with Apo A-I (*n* = 440) and Apo B (*n* = 255) were identified (Supplementary Table 4, available as Supplementary data at *IJE* online) using a recent GWAS of these apolipoproteins from UKBB^22^ and all these variants were independent based on *r*^2^ < 0.001 using a reference panel of Europeans from the 1000 genomes project. Respectively, 416 and 234 variants were available in the HUNT data set to examine the causal associations of these apolipoproteins with eGFRcrea.

To examine the causal effect of kidney-function markers (eGFRcrea, eGFRcys and UACR) on serum lipids, we obtained the marker-specific genome-wide significant and independent genetic variants from recently published GWASs. An instrument of 308 eGFRcrea-associated variants (Supplementary Table 5, available as Supplementary data at *IJE* online) was obtained from a recent CKD trans-ancestry GWAS analysis of 1 046 070 individuals.^17^ Out of these, 307 and 303 were available in the HUNT and UKBB, respectively. For eGFRcys, the 5 independent variants (Supplementary Table 5, available as Supplementary data at *IJE* online) were selected from the CKD genetic consortium GWAS of 32 834 individuals of European ancestry^23^ and 61 variants associated with UACR (Supplementary Table 5, available as Supplementary data at *IJE* online) were obtained using a GWAS of 547 361 individuals of European ancestry (including UKBB).^24^ All these variants were available in HUNT and UKBB.

### Mendelian Randomization

**Figure 1:**
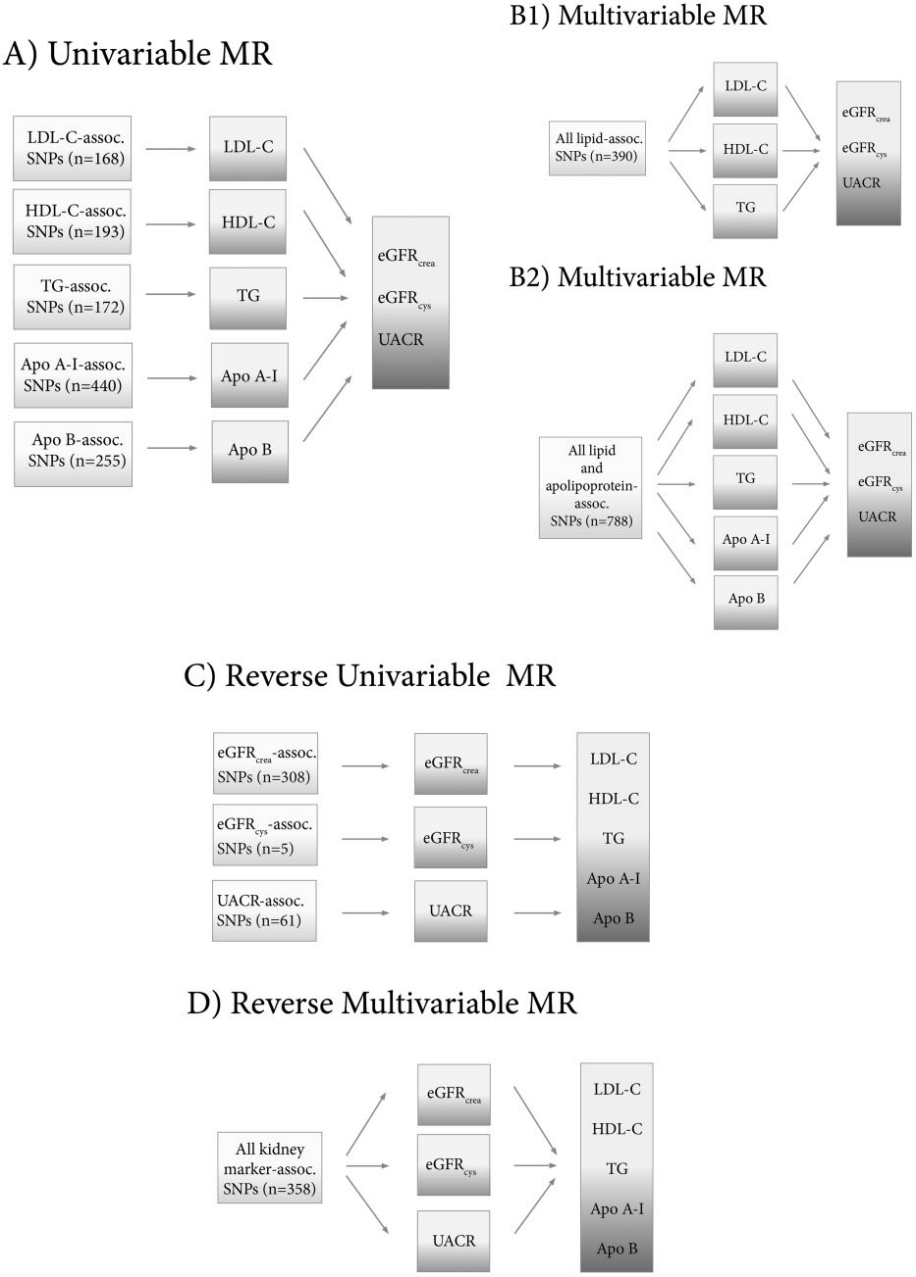
Schematic presentation of (A) univariable; (B) multivariable (with three lipid traits as B1 and five lipid and apolipoprotein traits as B2); (C) reverse univariable; and (D) reverse multivariable Mendelian randomization. HDL-C, high-density lipoprotein cholesterol; LDL-C, low-density lipoprotein cholesterol; TG, triglycerides; Apo A-I, apolipoprotein A-I; Apo B, apolipoprotein B; eGFRcrea, estimated glomerular-filtration rate based on creatinine measurements; eGFRcys, estimated glomerular-filtration rate based on cystatin C measurements; UACR, urinary albumin-to-creatinine ratio; SNP, single-nucleotide polymorphism; assoc., associated.

For univariable MR (Figure 1A), the causal effects of lipid fractions (using genetic variants associated with the lipid trait under study as instruments) on eGFRcrea, eGFRcys and UACR were estimated using inverse variance weighted (IVW) MR implemented in the TwoSampleMR R package.^25^ Multivariable MR (Figure 1B) is an MR extension analogous to assessing the effect of several treatments independently in one randomized control trial.^26^ For this method, the genetic instrument does not need to be exclusively associated with a single risk factor, but with a set of measured risk factors, although it must still satisfy equivalent instrumental-variable assumptions.^27^ Thus, the method can be applied for multiple genetic variants (not necessarily related to every exposure in the model) and several causally dependent or independent^26^ exposures in an instrumental-variable analysis to disentangle the direct causal effect of each risk factor included in the model. We applied this method using all lipid-associated variants (Figure 1B1; *n* = 390 for HUNT and UKBB) as instruments to estimate the independent effects of LDL-C, HDL-C and TG on kidney function, and all lipid- and apolipoprotein-associated variants (*n* = 788) as instruments to additionally estimate the independent effect of Apo A-I and Apo B (Figure 1B2, although the latter analysis carries the risk of bias due to sample overlap, as detailed below). Similarly, multivariable MR was used to disentangle the independent effect of each lipid exposure while controlling for genetic predisposition to body mass index (BMI), hypertension and type 2 diabetes in the model (instrument in Supplementary Table 6, available as Supplementary data at *IJE* online). The Apo A-I and Apo B instruments were tested against eGFRcrea in HUNT participants and for all three kidney markers in UKBB, although the latter analysis could be biased due to complete sample overlap (analysis summary outlined in Supplementary Table 7, available as Supplementary data at *IJE* online) between exposure and outcome data.^28^^,^^29^ Reverse-MR analysis (Figure 1C) was performed to investigate whether renal function, as represented by eGFRcrea, eGFRcys and kidney damage as represented by UACR may influence lipid or apolipoprotein traits (Supplementary Table 7, available as Supplementary data at *IJE* online). In an additional analysis, multivariable MR analysis (using 358 variants) was also used to assess the causal effect of individual kidney markers on lipid or apolipoprotein traits while controlling for the genetic predisposition to the other kidney markers (Figure 1D, details in Supplementary Methods and Supplementary Results, available as Supplementary data at *IJE* online). As explained in the Supplementary Methods, available as Supplementary data at *IJE* online, we put less emphasis on that analysis, as two of the kidney-function markers (eGFRcrea and eGFRcys) are not independent risk factors, but rather two ways of estimating the same underlying physiological trait, GFR. Details of other sensitivity analyses including MR-Egger, weighted median MR and MR with Steiger-filtering are provided in the Supplementary Methods section, available as Supplementary data at *IJE* online. All causal estimates represent the SD-unit change [with 95% confidence interval (CI)] in the outcome per 1-SD increase in the exposure, except for univariable MR estimates of causal effects of eGFR, which are reported per 1-unit increase in log-transformed eGFR. Random-effects meta-analysis was applied to calculate the combined MR estimates from HUNT and UKBB. Heterogeneity of summary estimates was estimated using Cochran’s Q test.

**Figure 2:**
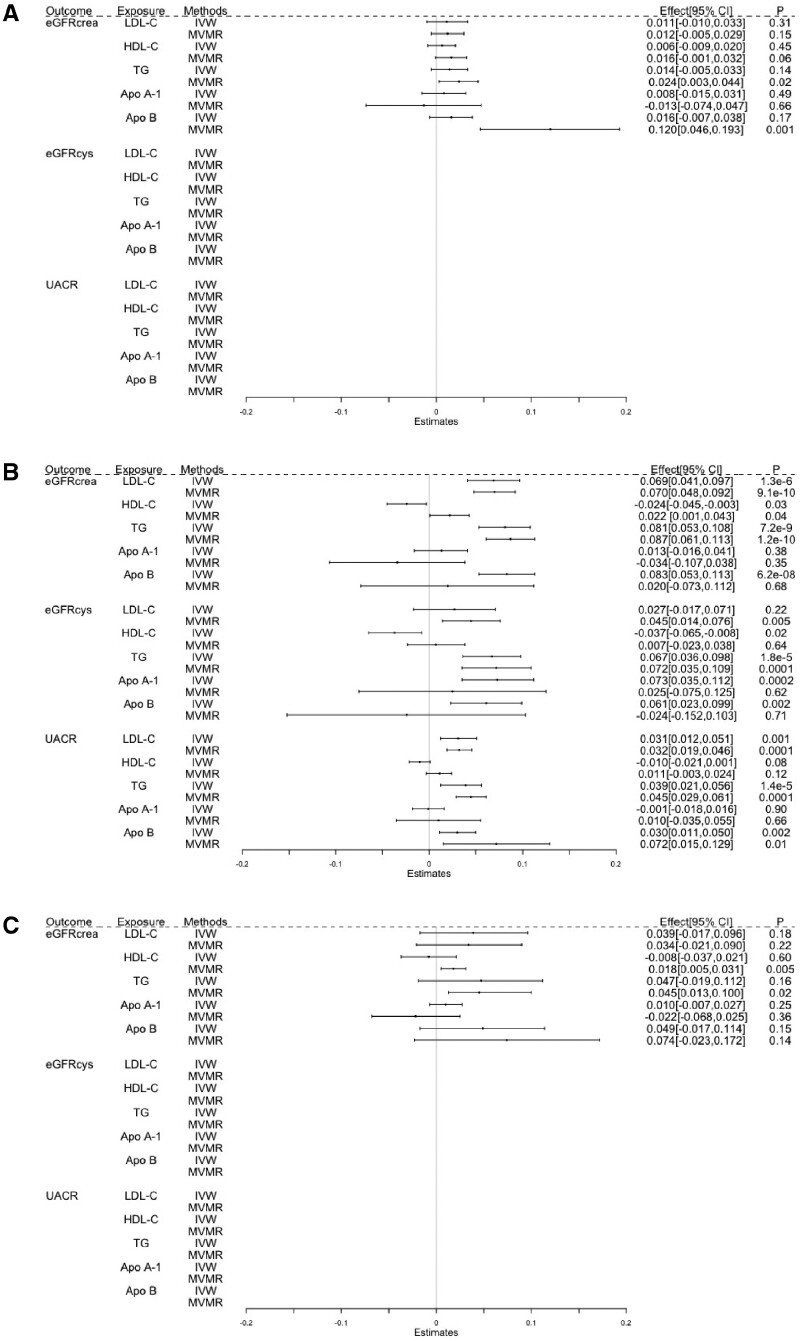
Estimated causal effects of lipids and apolipoproteins on kidney-function markers using univariable and multivariable MR, presented as the SD-unit change in kidney marker per genetically predicted 1-SD change in serum lipid and apolipoprotein levels. HDL-C, high-density lipoprotein cholesterol; LDL-C, low-density lipoprotein cholesterol; TG, triglycerides; Apo A-I, apolipoprotein A-I; Apo B, apolipoprotein B; eGFRcrea, estimated glomerular-filtration rate based on creatinine measurements; eGFRcys, estimated glomerular-filtration rate based on cystatin C measurements; UACR, urinary albumin-to-creatinine ratio; IVW, inverse variance-weighted Mendelian randomization; MVMR, multivariable MR; HUNT, Trøndelag Health Study; UKBB, the UK Biobank.

## Results

Characteristics of the HUNT and UKBB study populations are provided in Supplementary Table 8A and the strength of the selected genetic instruments is provided in Supplementary Table 8B and the Supplementary Results, all available as Supplementary data at *IJE* online. The observational associations and phenotypic and genotypic correlation of serum lipids and apolipoproteins with kidney-function markers in HUNT and UKBB are presented in Supplementary Figures 2 and 3, available as Supplementary data at *IJE* online, respectively.

## Associations of genetically predicted lipid and apolipoprotein levels with kidney function

### Univariable MR

Genetically predicted higher LDL-C was not convincingly associated with eGFR; eGFRcrea was 0.04 SD (95% CI: –0.02, 0.10; *P* = 0.18) higher and eGFRcys was 0.03 SD (95% CI: –0.02, 0.07; *P* = 0.22) higher per 1-SD higher genetically predicted LDL-C (Figure 2 and Supplementary Table 9A, available as Supplementary data at *IJE* online). However, estimates for eGFRcrea differed between HUNT and UKBB, with a stronger effect estimate of 0.07 SD (95% CI: 0.04, 0.10; *P* = 1.3 × 10^–6^) observed in UKBB. There was a weak UACR increase of 0.03 SD (95% CI: 0.01, 0.05; *P* = 0.001) per 1-SD higher genetically predicted LDL-C.

Genetically predicted higher HDL-C was not strongly associated with eGFR; a weak association of –0.04 SD (95% CI: –0.06, –0.01; *P* = 0.02) eGFRcys per 1-SD higher HDL-C was not supported by analysis of eGFRcrea (–0.01 SD; 95% CI: –0.04, 0.02; *P* = 0.60) or UACR (–0.01 SD; 95% CI: –0.02, 0.001; *P* = 0.08).

There was some evidence of a modest causal effect of TG on eGFR; each 1-SD higher genetically predicted TG increased eGFRcrea by 0.05 SD (95% CI: –0.02, 0.11; *P* = 0.16) and eGFRcys by 0.07 SD (95% CI: 0.04, 0.10; *P* = 1.8 × 10^–5^). The estimate for eGFRcrea was stronger in UKBB (0.08 SD; 95% CI: 0.05, 0.11; *P* = 7.2 × 10^–9^). Each 1-SD higher genetically predicted TG was also associated with 0.04 SD (95% CI: 0.02, 0.06; *P* = 1.4 × 10^–5^) higher UACR.

Genetically predicted higher Apo A-I was associated with increased eGFRcys (0.07 SD; 95% CI: 0.03, 0.11; *P* = 0.0002), but not eGFRcrea and UACR. Genetically predicted higher Apo B was weakly associated with higher eGFRcrea (0.05 SD; 95% CI: –0.02, 0.11; *P* = 0.15), with a stronger estimate in UKBB (0.08 SD; 95% CI: 0.05, 0.11; *P* = 6.2 × 10^–8^), where genetically predicted higher ApoB was also associated with slightly higher eGFRcys (0.06 SD; 95% CI: 0.02, 0.10; *P* = 0.002) and UACR (0.03 SD; 95% CI: 0.01, 0.05; *P* = 0.002).

### Multivariable MR

The associations of genetically predicted LDL-C and TG with kidney-function markers were similar in multivariable as in univariable MR (Figure 2). In contrast to the univariable MR analysis, multivariable MR showed a weak positive association between genetically predicted HDL-C and eGFRcrea (0.02 SD; 95% CI: 0.005, 0.03; *P* = 0.005), without corresponding associations with eGFRcys or UACR. Multivariable MR yielded little evidence of associations between genetically predicted Apo A-I and kidney-function markers. For genetically predicted Apo B, the positive association with eGFR observed using univariable MR was not seen in multivariable MR analysis, except for a positive association with eGFRcrea in HUNT (0.12 SD; 95% CI: 0.05, 0.19; *P* = 0.001) that we did not observe in UKBB. The association between genetically predicted Apo B and UACR was similar in multivariable as in univariable MR.

### Sensitivity analyses

Sensitivity analyses generally yielded similar estimates to the main analysis, with little evidence of directional pleiotropy (Supplementary Table 9A, available as Supplementary data at *IJE* online). Sensitivity analyses using more independent SNPs (pruning for *r*^2^ = 0.001) were consistent with the main results (Supplementary Table 9B, available as Supplementary data at *IJE* online). Although the TG-increasing genetic risk score (GRS) (details in Supplementary Methods, available as Supplementary data at *IJE* online) was associated with BMI (Supplementary Figure 4C_HUNT, available as Supplementary data at *IJE* online), the associations of genetically predicted TG with kidney-function markers were not attenuated after adjustment for the genetic predisposition to higher BMI, hypertension and type 2 diabetes (Supplementary Table 10, available as Supplementary data at *IJE* online).

## Associations of genetically predicted levels of kidney-function markers with lipid and apolipoprotein levels

**Figure 3:**
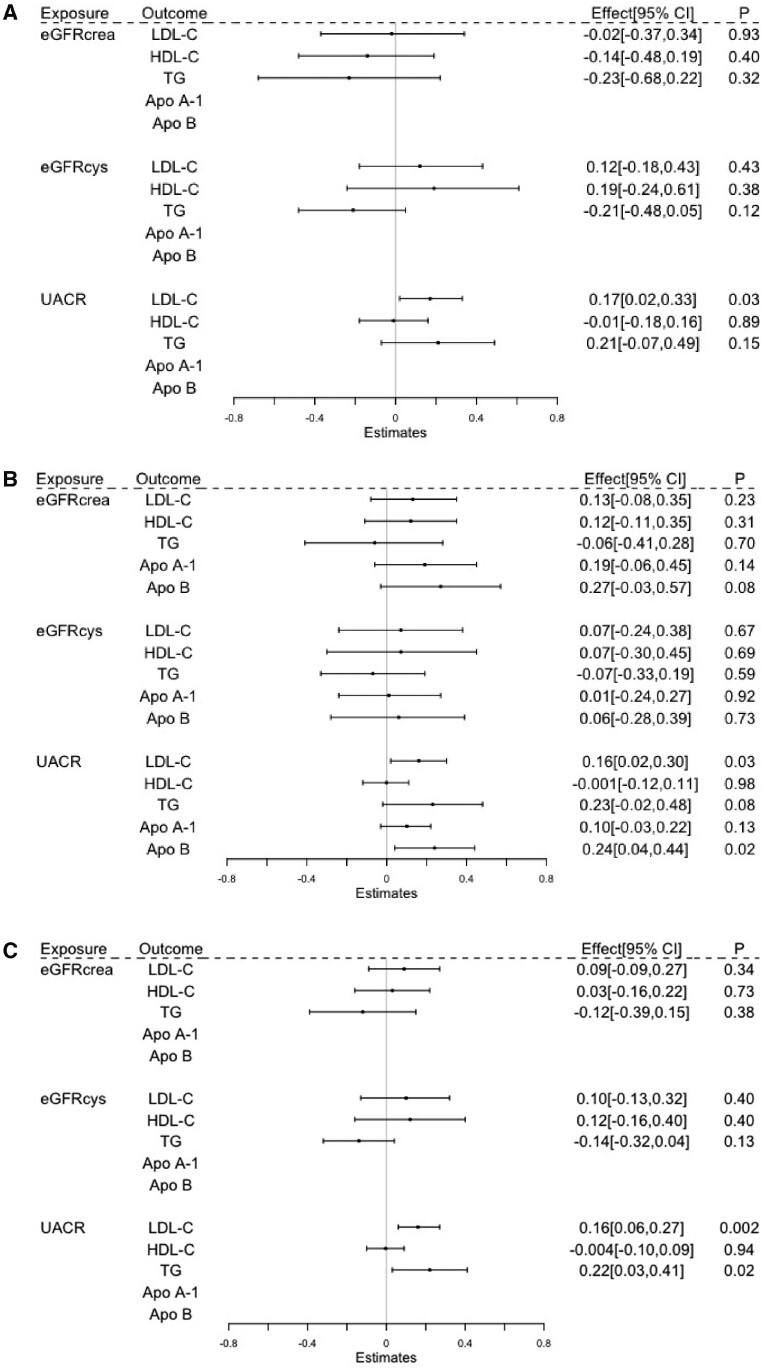
The inverse variance-weighted (IVW) estimated causal effects of kidney-function markers on lipid and apolipoprotein traits, indicated as the SD-unit change in lipid and apolipoprotein traits per genetically predicted 1-SD change in UACR and per 1-unit change in log(eGFRcrea) or log(eGFRcys). HDL-C, high-density lipoprotein cholesterol; LDL-C, low-density lipoprotein cholesterol; TG, triglycerides; Apo A-I, apolipoprotein A-I; Apo B, apolipoprotein B; eGFRcrea, estimated glomerular-filtration rate based on creatinine measurements; eGFRcys, estimated glomerular-filtration rate based on cystatin C measurements; UACR, urinary albumin-to-creatinine ratio; HUNT, Trøndelag Health Study; UKBB, the UK Biobank.

Univariable MR analysis provided little evidence of effects of kidney function measured by eGFRcrea or eGFRcys on serum lipids or apolipoproteins (Figure 3). For example, changes in lipids per 1-unit higher genetically predicted log(eGFRcrea) were 0.09 SD (95% CI: –0.09, 0.27; *P* = 0.34) for LDL-C, 0.03 SD (95% CI: –0.16, 0.22; *P* = 0.73) for HDL-C, –0.12 SD (95% CI: –0.39, 0.15; *P* = 0.38) for TG, 0.19 SD (95% CI: –0.06, 0.45; *P* = 0.14) for Apo A-1 and 0.27 SD (95% CI: –0.03, 0.57; *P *= 0.08) for Apo B (Figure 3). In general, sensitivity analyses yielded similar results and there was little sign of directional pleiotropy. However, there was some evidence of a causal role of genetically raised eGFRcrea in reducing TG levels after Steiger-filtering in sensitivity analysis (Supplementary Table 11, available as Supplementary data at *IJE* online).

Genetically predicted higher UACR was associated with increased LDL-C (0.16 SD; 95% CI: 0.06, 0.27; *P* = 0.002), TG (0.22 SD; 95% CI: 0.03, 0.41; *P *= 0.02) and Apo B (0.24 SD; 95% CI: 0.04, 0.44; *P* = 0.02), but not HDL-C (–0.004 SD; 95% CI: –0.10, 0.09; *P *= 0.94) and Apo A-I (0.10 SD; 95% CI: –0.03, 0.22; *P *= 0.13). Findings from all sensitivity approaches were consistent in direction and there was little sign of directional pleiotropy (Supplementary Table 11, available as Supplementary data at *IJE* online).

## Discussion

In this MR study, we examined whether the previously observed associations of serum lipid and apolipoprotein levels with kidney-function markers may be causal. Our meta-analysis of HUNT and UKBB yielded causal estimates that are not compatible with serum lipids or apolipoproteins having strong causal effects on kidney function, as indicated by eGFRcrea and eGFRcys. However, there was some, but inconsistent, evidence that higher levels of atherogenic lipids indicated by LDL-C, TG and Apo B may weakly increase both eGFR (suggesting better kidney function) and UACR (suggesting kidney stress or damage). Our reverse-MR analysis provided no clear evidence that eGFR may influence lipid or apolipoprotein traits, but processes leading to higher UACR may lead to more atherogenic lipids including higher LDL-C, TG and Apo B.

A recent MR study by Lanktree *et al.*^18^ (with no sample overlap with UKBB and HUNT) using 183 lipid instruments explaining 8.9% variation in HDL-C concluded a positive causal association of HDL-C with eGFRcrea in both univariable and multivariable MR analyses, and a negative association with UACR. However, another previous MR analysis with 68 ‘lead’ HDL-C-associated variants (explaining 6.6% variation in HDL-C)^30^ along with our findings using an improved lipid instrument (explaining 14.6% variation for HDL-C) does not provide convincing support for a causal role of HDL-C on these kidney markers. Our results for HDL-C differed between eGFRcrea and eGFRcys, and between univariable and multivariable MR, but neither analysis suggested substantial effects. Concordantly, results of a recent clinical trial of niacin for improving eGFR^20^ also challenge the suggested effect of HDL-C on eGFR.

We obtained some evidence supporting weak causal effects of higher LDL-C, TG and ApoB on higher eGFR. These observations are compatible with the findings reported by Lanktree *et al.*^18^ In line with our findings for UACR, a recent Chinese study showed that each 1-mmol/L increase in the genetically predicted TG increased the risk of CKD by 5%.^31^ It seems unexpected that higher levels of atherogenic lipids should both increase UACR (which may indicate kidney stress or damage) and increase glomerular filtration (which may be considered as a sign of better kidney function). However, it is possible that higher eGFR associated with atherogenic lipids indicates glomerular hyperfiltration that may occur in people with cardiometabolic conditions.^32^ Collectively, these findings suggest a complex causal role of atherogenic lipids in kidney function and disease that requires further investigation.

Our reverse-MR analyses indicated no substantial causal effects of eGFR on HDL-C and LDL-C. The main analysis also did not indicate a causal effect of eGFR on TG; however, there was some evidence for a causal role of genetically raised eGFRcrea in reducing TG levels in both HUNT and UKBB in the Steiger-filtered sensitivity analysis. This finding is consistent with observational studies of the association between eGFRcrea and TG.^33–36^ Consistently with observational studies,^13^^,^^14^^,^^37^^,^^38^ we observed substantial evidence for a causal effect of processes leading to higher UACR in increasing levels of atherogenic lipids. Studies of dyslipidemia in nephrotic syndrome, in which urinary albumin excretion is strongly increased,^39^ may be relevant for understanding these findings. Patients with nephrotic syndrome have higher circulating levels of cholesterol, TG and Apo B-containing lipoproteins, whereas the concentrations of HDL-C and Apo A-containing lipoproteins are comparable to those in healthy individuals.^40^^,^^41^ Impaired urinary clearance, dysfunction of hepatic LDL receptors and hepatic lipase may contribute to the dyslipidemia seen in this syndrome.^42^

The strengths of our study include the use of two large data sets (UKBB and HUNT), updated genetic instruments for lipid and apolipoprotein traits, inclusion of eGFRcys as a superior alternative to eGFRcrea not influenced by muscle mass,^43^ reverse MR for kidney-function markers vs lipid and apolipoprotein traits, and a range of sensitivity-analysis approaches including multivariable MR-Egger and Steiger-filtered MR to increase the reliability of the causal estimates. One limitation is that, when studying the effects of multiple correlated exposures using multivariable MR, use of SNPs that are associated with multiple exposures can lead to exposures that are strongly predicted individually but only weakly predicted by those SNPs conditional upon the other exposures included and thus leading to weak instrument bias.^44^ Another limitation of multivariable MR is that the method can deal with ‘measured’ pleiotropic associations only and is unable to deal with unmeasured or unknown counterparts. Multivariable MR-Egger^45^ can address the pleiotropy, but the orientation of the effect sizes is still a methodological challenge for this approach. The non-fasting state of the lipid measurements in both HUNT and UKBB may have influenced the TG levels, but is unlikely to have substantially influenced our estimates.^46^ Notably, the analysis was performed using data sets representative of the general population (with >90% of the subjects having normal kidney markers), so the changes in lipids associated with advanced stages of CKD cannot be answered by our study. We acknowledge that HUNT is one of the study cohorts in the lipid GWAS used here but it makes up 1.6% of the total sample and thus cannot introduce substantial bias due to participant overlap.^28^ For the UACR instrument, as UKBB contributes a major share (79.7%) of the GWAS,^24^ the reverse-MR results for UKBB may suffer from bias due to sample overlap,^28^^,^^29^ although comparable effect estimates from HUNT (as an independent cohort) improve the reliability of causal estimates from meta-analysis. Bias due to sample overlap could also influence the Apo A-I and Apo B results using both exposure and outcome information from UKBB. The discrepancy between UKBB and HUNT estimates for the associations of genetically predicted LDL-C and TG with eGFRcrea was unexpected, but collider bias due to low participation could have influenced the UKBB results.^47^

Collectively, our MR estimates supported that serum lipid and apolipoproteins levels do not cause substantial changes in kidney function. The possible weak causal effects of atherogenic lipids on higher eGFR and UACR need further replication and understanding. For the reverse causal direction, there was no consistent evidence that eGFR may influence serum lipid or apolipoprotein levels, but processes leading to higher UACR may cause an increase in atherogenic lipids indicated by higher LDL-C, TG and Apo B levels.

## Supplementary data

Supplementary data are available at *IJE* online.

## Funding

Dr Zhang is supported by the Vice-Chancellor fellowship and Dr Sanderson was supported by Medical Research Council (MC_UU_00011/1) during the study. Professors Davey Smith and Gaunt work in the Medical Research Council Integrative Epidemiology Unit at the University of Bristol (MC_UU_00011/1&4) and have received research funding from GlaxoSmithKline and Biogen.

## Ethics approval

The HUNT study was approved by the Central Norway Regional Committee for Medical and Health Research Ethics (REC Central no. 2015/1188) and written informed consent was given by all participants. The UK Biobank study has ethical approval from the North West Multi-center Research Ethics Committee (MREC).

## Data availability

The data underlying this article will be shared on reasonable request to the corresponding author.

## Supplementary Material

dyab014_Supplementary_Data
