# Supplementary material for: The causal effects of serum lipids and apolipoproteins on kidney function: multivariable and bidirectional Mendelian-randomization analyses
Source: Int J Epidemiol. 2021 Jun 21;50(5):1569–79. doi: 10.1093/ije/dyab014 (PMC8580277; doi:10.1093/ije/dyab014)
Supplement: dyab014_Supplementary_Data [file dyab014_supplementary_data.zip › ije-2020-02-0192-File012.pdf]

Supplementary Figures

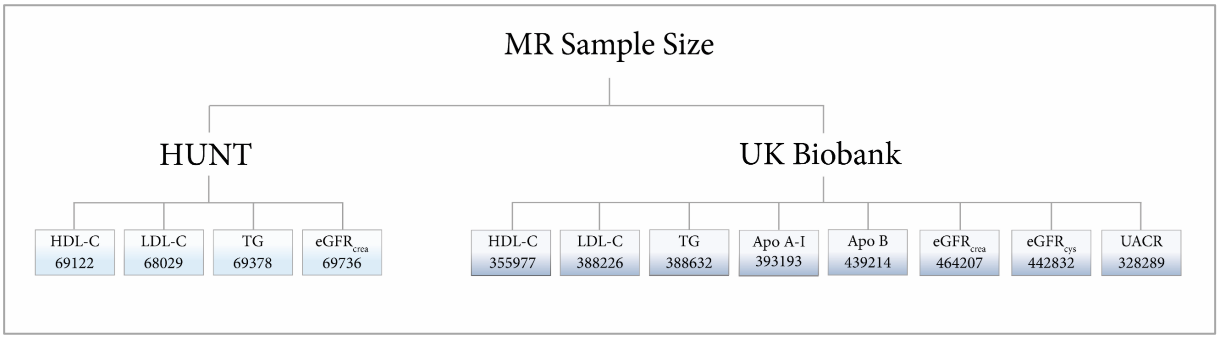

Figure S1: Flow chart to show the number of subjects used in creating summary data for each MR analysis. Abbreviations: HDL-C: high-density lipoprotein cholesterol; LDL-C: low-density lipoprotein cholesterol; TG: triglycerides; Apo A-I: apolipoprotein A-I; Apo B: apolipoprotein B; eGFRcrea: estimated glomerular filtration rate based on creatinine measurements; eGFRcys: estimated glomerular filtration rate based on cystatin C measurements; UACR: urinary albumin to creatinine ratio; HUNT: Trøndelag Health Study; UKBB: the UK Biobank.

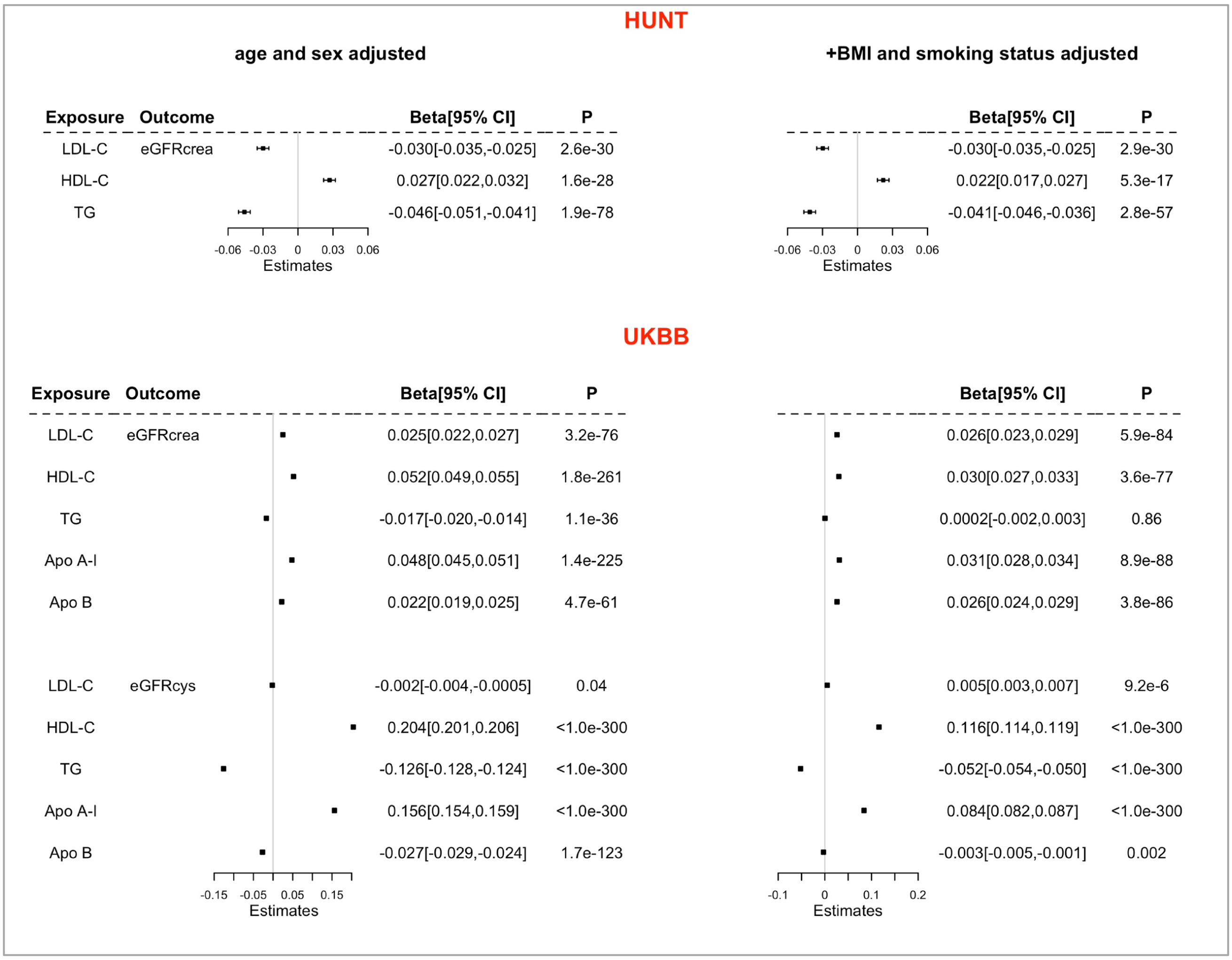

Figure S2: Observational analysis for SD change in eGFRcrea and eGFRcys per one SD increase in lipid and apolipoprotein traits in HUNT (Top panel) and UKBB (Bottom panel), adjusted for age, sex, BMI and smoking status. Abbreviations: HDL-C: high-density lipoprotein cholesterol; LDL-C: low-density lipoprotein cholesterol; TG: triglycerides; Apo A-I: apolipoprotein A-I; Apo B: apolipoprotein B; eGFRcrea: estimated glomerular filtration rate based on creatinine measurements; eGFRcys: estimated glomerular filtration rate based on cystatin C measurements; BMI: body mass index; HUNT: Trøndelag Health Study; UKBB: the UK Biobank.

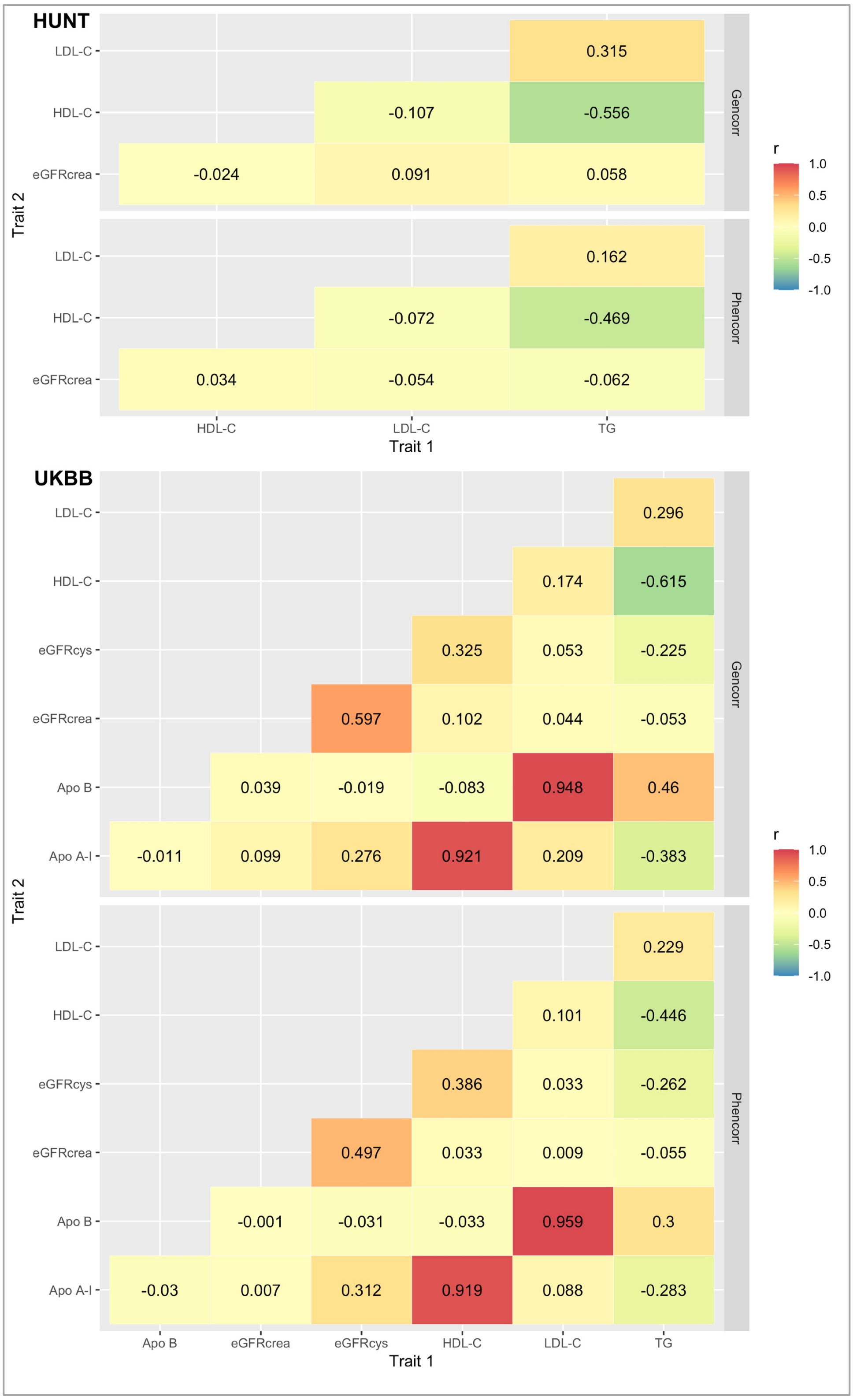

Figure S3: Heatmap to show the phenotypic and genotypic correlation between lipid (LDL-C, HDL-C, TG) and apolipoprotein (Apo A-I, Apo B) traits with two kidney function markers (eGFRcrea, eGFRcys) using HUNT (top panel) and UKBB (bottom panel) data sets. Abbreviations: HDL-C: high-density lipoprotein cholesterol; LDL-C: low-density lipoprotein cholesterol; TG: triglycerides; Apo A-I: apolipoprotein A-I; Apo B: apolipoprotein B; eGFRcrea: estimated glomerular filtration rate based on creatinine measurements; eGFRcys: estimated glomerular filtration rate based on cystatin C measurements; HUNT: Trøndelag Health Study; UKBB: the UK Biobank.

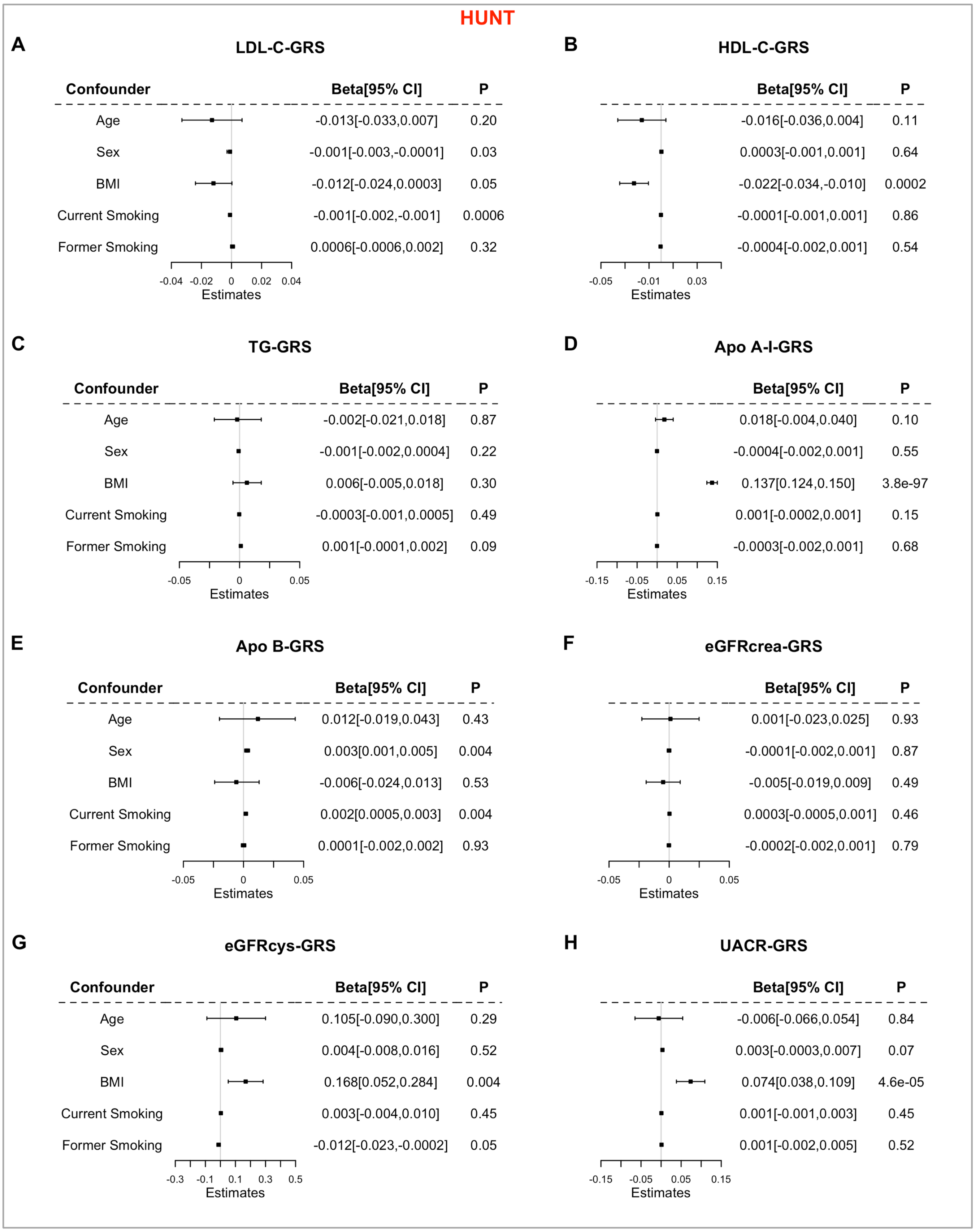
HUNT
A
LDL-C-GRS
Confounder
Beta[95% CI]
P
Age -0.013[-0.033,0.007] 0.20
Sex -0.001[-0.003,-0.0001] 0.03
BMI -0.012[-0.024,0.0003] 0.05
Current Smoking -0.001[-0.002,-0.001] 0.0006
Former Smoking 0.0006[-0.0006,0.002] 0.32
-0.04 -0.02 0 0.02 0.04
Estimates
B
HDL-C-GRS
Beta[95% CI]
P
-0.016[-0.036,0.004] 0.11
0.0003[-0.001,0.001] 0.64
-0.022[-0.034,-0.010] 0.0002
-0.0001[-0.001,0.001] 0.86
-0.0004[-0.002,0.001] 0.54
-0.05 -0.01 0.03
Estimates
C
TG-GRS
Confounder
Beta[95% CI]
P
Age -0.002[-0.021,0.018] 0.87
Sex -0.001[-0.002,0.0004] 0.22
BMI 0.006[-0.005,0.018] 0.30
Current Smoking -0.0003[-0.001,0.0005] 0.49
Former Smoking 0.001[-0.0001,0.002] 0.09
-0.05 0 0.05
Estimates
D
Apo A-I-GRS
Beta[95% CI]
P
0.018[-0.004,0.040] 0.10
-0.0004[-0.002,0.001] 0.55
0.137[0.124,0.150] 3.8e-97
0.001[-0.0002,0.001] 0.15
-0.0003[-0.002,0.001] 0.68
-0.15 -0.05 0.05 0.15
Estimates
E
Apo B-GRS
Confounder
Beta[95% CI]
P
Age 0.012[-0.019,0.043] 0.43
Sex 0.003[0.001,0.005] 0.004
BMI -0.006[-0.024,0.013] 0.53
Current Smoking 0.002[0.0005,0.003] 0.004
Former Smoking 0.0001[-0.002,0.002] 0.93
-0.05 0 0.05
Estimates
F
eGFRcrea-GRS
Beta[95% CI]
P
0.001[-0.023,0.025] 0.93
-0.0001[-0.002,0.001] 0.87
-0.005[-0.019,0.009] 0.49
0.0003[-0.0005,0.001] 0.46
-0.0002[-0.002,0.001] 0.79
-0.05 0 0.05
Estimates
G
eGFRcys-GRS
Confounder
Beta[95% CI]
P
Age 0.105[-0.090,0.300] 0.29
Sex 0.004[-0.008,0.016] 0.52
BMI 0.168[0.052,0.284] 0.004
Current Smoking 0.003[-0.004,0.010] 0.45
Former Smoking -0.012[-0.023,-0.0002] 0.05
-0.3 -0.1 0.1 0.3 0.5
Estimates
H
UACR-GRS
Beta[95% CI]
P
-0.006[-0.066,0.054] 0.84
0.003[-0.0003,0.007] 0.07
0.074[0.038,0.109] 4.6e-05
0.001[-0.001,0.003] 0.45
0.001[-0.002,0.005] 0.52
-0.15 -0.05 0.05 0.15
Estimates

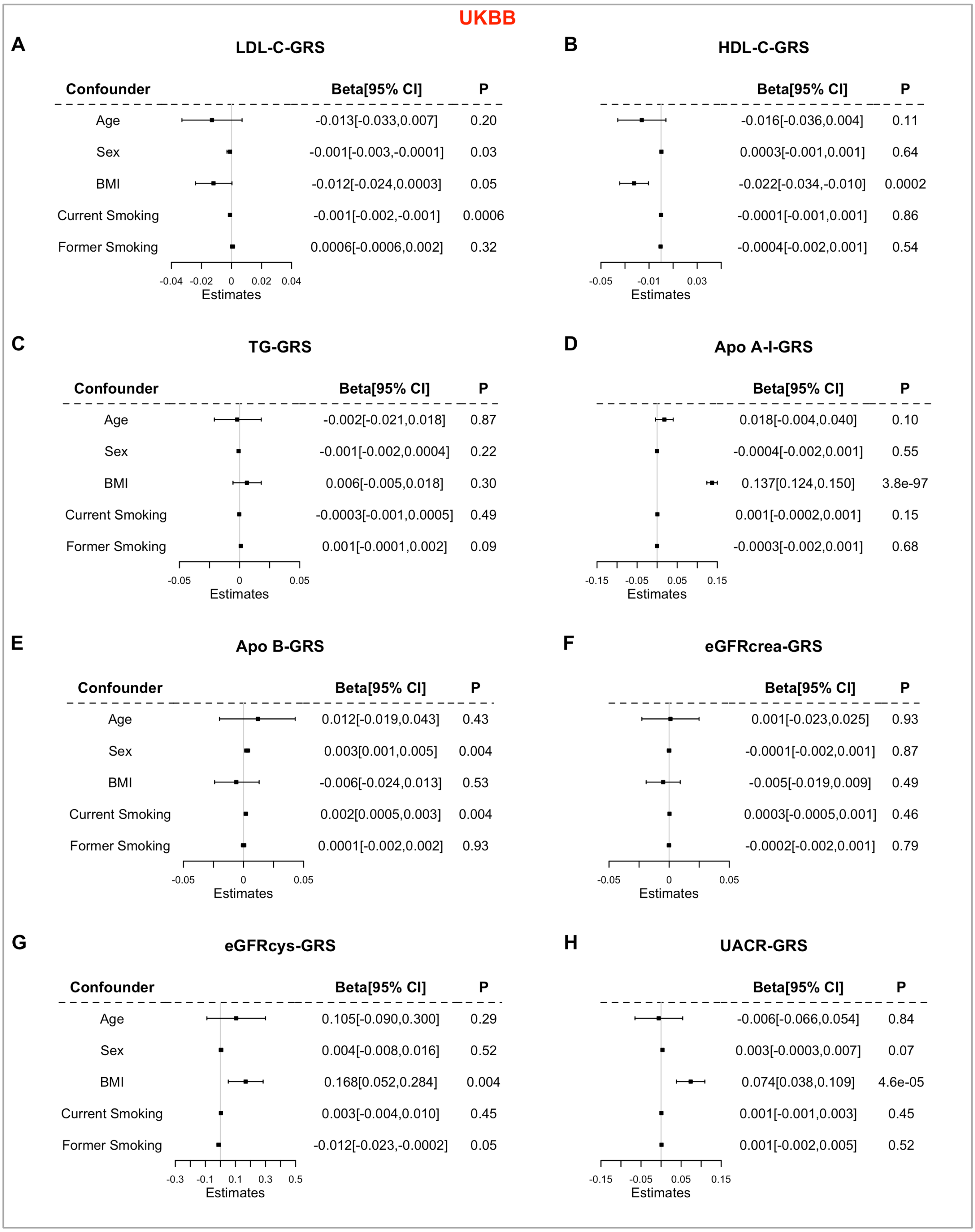

Figure S4: Change in individual phenotypes per 10 units increase in the unweighted genetic risk score in HUNT (top panel) and UKBB (bottom panel) participants. Abbreviations: BMI: body mass index; GRS: genetic risk score: HDL-C: high-density lipoprotein cholesterol; LDL-C: low-density lipoprotein cholesterol; TG: triglycerides; Apo A-I: apolipoprotein A-I; Apo B: apolipoprotein B; eGFRcrea: estimated glomerular filtration rate based on creatinine measurements; eGFRcys: estimated glomerular filtration rate based on cystatin C measurements; UACR: urinary albumin to creatinine ratio; HUNT: Trøndelag Health Study; UKBB: the UK Biobank.
