# Supplementary material for: The causal effects of serum lipids and apolipoproteins on kidney function: multivariable and bidirectional Mendelian-randomization analyses
Source: Int J Epidemiol. 2021 Jun 21;50(5):1569–79. doi: 10.1093/ije/dyab014 (PMC8580277; doi:10.1093/ije/dyab014)
Supplement: dyab014_Supplementary_Data [file dyab014_supplementary_data.zip › ije-2020-02-0192-File013.docx]

**SUPPLEMENTARY MATERIAL**

**SUPPLEMENTARY METHODS**

**HUNT**

HUNT is a series of general health surveys of the adult population of the demographically stable Trøndelag county, Norway, as detailed elsewhere^1^. So far four health surveys have been conducted, HUNT1 (1984–1986), HUNT2 (1995–1997), HUNT3 (2006–2008) and HUNT4 (2017-2019)^2^. Our study includes participants from the HUNT2 and HUNT3 surveys with complete genotype and phenotype data (numbers in Supplementary Figure S1).

Serum creatinine levels were measured in fresh serum samples using the Jaffé method and calibrated to isotope-dilution mass spectroscopy level. Estimated glomerular filtration rate using serum creatinine (eGFRcrea) was estimated with the Chronic Kidney Disease Epidemiology consortium (CKD-EPI) formula^3^. Total cholesterol, high-density lipoprotein cholesterol (HDL-C) and triglycerides (TG) levels were measured in non-fasting serum samples using enzymatic colorimetric cholesterol esterase methods (Boehringer Mannheim, Mannheim, Germany) in HUNT2. In HUNT3, total cholesterol was measured by enzymatic cholesterol esterase methodology, HDL-C by accelerator selective detergent methodology, and TG by glycerol phosphate oxidase methodology. Low-density lipoprotein cholesterol (LDL-C) levels were calculated using the Friedewald formula^4^. Participants in HUNT with TG levels ≥4.5mmol/L (n=1349) were excluded for LDL-C calculation, as the Friedewald formula is not valid at higher TG levels. For the phenotype data, if a participant attended both HUNT2 and HUNT3 survey then measurements from HUNT2 were preferred over HUNT3 as the earlier measurement will likely be less influenced by age-related diseases.

DNA samples were available from more than 90% of subjects from HUNT2 and HUNT3 and were genotyped^5^ using one of the three different Illumina HumanCoreExome arrays: HumanCoreExome12 v1.0 (n= 7570), HumanCoreExome12 v1.1 (n=4960) and University of Michigan HUNT Biobank v1.0 (n=58041; HumanCoreExome*-*24 v1.0, with custom content). The genotypes from different arrays had quality control performed separately and the call rate of genotyped samples was >99%. Imputation was performed on samples of recent European ancestry using Minimac3 (v2.0.1, <http://genome.sph.umich.edu/wiki/Minimac3>)^6^ from a merged reference panel constructed from i) the Haplotype Reference Consortium panel (release version 1.1)^7^ and ii) a local reference panel based on 2,202 whole-genome sequenced HUNT participants^8^. Subjects included in the study were of European ancestry and had passed the quality control.

**UKBB**

We used a data set of 464,207 unrelated participants of European ancestry (Supplementary Figure S1) from the UK Biobank (UKBB). Serum creatinine levels were measured using enzymatic analysis on a Beckman Coulter AU5800 and the eGFRcrea values were estimated using the Chronic Kidney Disease Epidemiology Collaboration (CKD-EPI) formula^3^. Cystatin C (cys) was measured using latex enhanced immunoturbidimetric analysis on a Siemens ADVIA 1800, and these measurement were used to calculate the eGFRcys using the Chronic Kidney Disease Epidemiology Collaboration (CKD-EPI) formula^3^. Urinary albumin to creatinine ratio was calculated using albumin and creatinine levels measured photometrically on a Beckman Coulter analyzer^9^. Total cholesterol and TG were measured by enzymatic colorimeter method, HDL-C by enzyme immunoinhibition method, LDL-C by enzymatic protective selection method, and for apolipoprotein A-I (Apo A-I) and apolipoprotein B (Apo B) by immunoturbidimetric analysis on a Beckman Coulter AU5800.

The Affymetrix UK BiLEVE Axiom array was used to genotype the initial 50,000 participants and the Affymetrix UK Biobank Axiom® array was used to genotype rest of the subjects. The two arrays were extremely similar and quality control and imputation was conducted by collaborative group headed by the Wellcome Trust Centre for Human Genetics^10^.

**Genetic association data**

The genetic associations for lipids, apolipoproteins, eGFRcrea, eGFRcys and UACR (when used as exposures) were obtained from the GLGC^11^, UKBB^12^ and eGFRcrea^13^, eGFRcys^14^ and UAC^15^ GWASs, respectively. The associations between the genetic instruments and the outcomes were calculated using SAIGE v0.29^16^ linear mixed model adjusted for age, sex, genotyping batch, and genetic principal components (PCs) 1 to 4 in HUNT and using BOLT-LMM^17^ linear mixed models as an additive genetic model adjusted for age, sex, genotyping array, and genetic correlation matrix [<https://doi.org/10.5523/bris.pnoat8cxo0u52p6ynfaekeigi>] in the UKBB. Publicly available GWAS summary data for UACR (as outcome) were used from a recently published source^9^, where the UACR measurements were rank-based inverse normal transformed before running the GWAS.

**Reverse multivariable MR**

For the reverse multivariable MR analysis (as presented in Figure 1D), the univariable MR instruments for eGFRcrea^13^, eGFRcys^14^ and UACR^15^ summed up as 358 SNPs. However, only 166 of these variants were available in the referenced eGFRcys GWAS. Moreover, the units of measurement for the instrument-exposure association differed between the eGFRcrea and eGFRcys (log-transformed scale) and the UACR (inverse-normalized scale) GWASs. To keep the measurement scale consistent (inverse-normalized) across three phenotypes and to avoid the unnecessary loss of SNPs we used the same set of SNPs (n=358) as from the univariable MR analysis and obtained the instrument-exposure associations for both eGFRcrea and eGFRcys from UKBB. Thus, when interpreting the findings from this multivariable MR analyses of kidney function markers with lipid and apolipoprotein traits in UKBB, the possibility of sample overlap bias has to be taken into account. Further, eGFRcrea and eGFRcys are two estimates for the same underlying biological trait, eGFR. Thus, any differences in the estimated effect of eGFRcrea when controlling for the effect of eGFRcys, or *vice versa*, would likely be a result of the different properties of these two variables as being proxies for eGFR, rather than different biological effects of eGFRcrea and eGFRcys *per se*.

**Observational analysis**

In HUNT, we performed age- and sex-adjusted linear regression to estimate the observational difference (in SD units) in eGFRcrea per one SD higher HDL-C, LDL-C and TG. The associations were additionally adjusted for body mass index (BMI) and smoking status. Similar analysis was performed using 5 lipid and apolipoprotein traits (LDL-C, HDL-C, TG, Apo A-I and Apo B) with glomerular filtration rate traits (eGFRcrea and eGFRcys) using individual level data from UKBB. As the UACR information we used was from an already published GWAS^15^ rather than from individual-level data accessible to us, this and follow-up analyses in next sections were not performed for UACR.

**Phenotypic and genotypic correlations**

Using individual levels data, we calculated phenotypic and genotypic correlation between four traits (LDL-C, HDL-C, TG, eGFRcrea) from HUNT and 7 traits (LDL-C, HDL-C, TG, Apo A-I, Apo B, eGFRcrea, eGFRcys) from UKBB. The genetic correlations for HUNT were computed with the Genome-wide Complex Trait Analysis software (GCTA)^18^ version 1.93.2beta, by first computing genetic relationship matrix (GRM) on autosome variants with minimal allele frequency of at least 1%, and selecting unrelated individuals with a genomic relationship less than 0.025. Then we used the reml-bivar method^19^ from GCTA on the GRM and the phenotype values to estimate genetic correlations between each pair of traits.

To estimate the genetic correlation for UKBB traits, we used a recent method based on LD score regression^20, 21^. This method uses the cross‐products of summary test statistics from two GWASs and regresses them against a measure of how much variation each SNP tags (its LD score). Variants with high LD scores are more likely to contain more true signals and thus provide a greater chance of overlap with genuine signals between GWASs. The LD score regression method uses summary statistics from the GWAS meta‐analysis of the seven phenotypes, calculates the cross‐product of test statistics at each SNP, and then regresses the cross‐product on the LD score.

For phenotypic correlations in both data sets, Pearson correlations were computed in R on the same set of individuals as the genetic correlations. All correlation (between and within traits) values were plotted as heatmaps using ggplot function in R.

**Testing MR assumptions using individual-level data**

Three key assumptions for MR are that the genetic variants must be associated with the exposure, that the instrument should not influence the outcome except through the exposure, and that there should be no confounders of the genetic variants-outcome association^22^.

To test the first assumption, associations of genetic variants with the exposures were estimated using regression models. The unweighted genetic risk scores (GRS) using available lipid, apolipoprotein and kidney function marker traits-associated variants were calculated as described elsewhere^23^. To obtain the F-statistics, this GRS was regressed with respective phenotype measurements using individual level data from HUNT and UKBB. Moreover, for lipids we also estimated the Sanderson–Windmeijer conditional F-statistic^24^ for the multivariable MR analysis. For the latter estimation, weighted GRS was calculated (as described elsewhere^23^) using all 390 lipid-associated variants along with the effect estimates for LDL-C, HDL-C and TG from the respective GWAS and additionally all lipid and apolipoprotein traits with GWAS measures from respective studies for UKBB only. These weighted GRSs were then regressed against the individual phenotypes, using models described elsewhere^25^. We used this method to estimate the strength of the instrument for each individual lipid or apolipoprotein trait given the other lipid traits. As a rule-of-thumb, an F-statistic of at least 10 is commonly considered as indication of a sufficiently strong instrument to avoid weak instrument bias.

We used linear and logistic regression models to examine whether the the unweighted GRSs (calculated using available lipid-associated variants- and kidney markers-associated variants) were associated with potential confounders for the lipids-kidney markers associations: age, sex, BMI and smoking.

**Sensitivity analysis**

To examine whether the estimates may be influenced by horizontal pleiotropy, i.e. that the instrument influences the outcome through pathways other than through the exposure, we applied weighted median (WM) regression^26^, MR-Egger regression^27^ and an extension of MR-Egger for multivariable MR^28^. The WM method can provide valid causal estimates even if up to 50% of the information comes from invalid instrumental variables^26^ and MR-Egger provides valid estimates even when all the genetic variants are invalid instrumental variables as long as the InSIDE (Instrument Strength Independent of Direct Effect) assumption is satisfied ^27^. In the analyses using multiple related phenotypes with shared genetic predictors, as is the case with lipid traits, multivariable MR-Egger is a preferred sensitivity analysis, but its results should be interpreted carefully as the orientation of the direction effects is still a methodological issue^28^.

Considering the directionality assumptions of MR, it is expected that the selected SNPs are primarily associated with the exposure and any association with the outcome is only via the exposure of interest. However, in a selected instrument, some of the SNPs may be primarily associated with the outcome instead of the exposure. Such SNPs might have reached genome-wide significance with the exposure due to large sample size and thus have been mistakenly selected as exposure-related instruments. Use of such SNPs as exposure-associated instruments can introduce a violation of the directionality assumption. The Steiger filtering approach^29^, implemented in the TwoSampleMR R package^30^, was used to estimate the corrected causal association of the exposure of interest on the outcome. The approach is sensitive to differences in the power of the GWAS for the exposure and the outcome and infers the causal direction between phenotypes using a simple inequality that is estimated using the correlation of SNPs with exposure and outcome. A stronger correlation of any SNPs with the outcome than the exposure indicates that the tested SNP is primarily affecting the outcome. In Steiger filtering, such SNPs were removed, and the MR analyses were repeated to calculate IVW, WM and MR-Egger estimates.

**SUPPLEMENTARY RESULTS**

**Observational analyses**

Age- and sex-adjusted observational analyses in HUNT showed that all three lipid traits, LDL-C, HDL-C and TG, were associated with eGFRcrea though the observational estimates were small. The SD change (95% CI) in eGFRcrea per 1SD higher lipids was -0.030 (-0.035,-0.025) for LDL-C, 0.027 (0.022,0.032) for HDL-C and -0.046 (-0.051,-0.041) for TG. Additional adjustment for BMI and smoking status modestly attenuated these estimates (Supplementary Figure S2, top panel).

In UKBB, LDL-C was positively associated with eGFRcrea, but the estimate for the association with eGFRcys was close to the null. HDL-C was positively associated with eGFRcrea and eGFRcys, and TG negatively associated with eGFRcys. Apo A-I was positively associated with eGFRcrea and eGFRcys, whereas Apo B was positively associated with eGFRcrea but negatively with eGFRcys (Supplementary Figure S2, bottom panel).

**Phenotypic and genotypic correlations**

In HUNT, there was a positive phenotypic correlation between eGFRcrea and HDL-C and negative correlations of eGFR with LDL-C and TG, with opposite direction for the genotypic correlations (Supplementary Figure S3, top panel). In UKBB, there were positive phenotypic and genotypic correlations of LDL-C, HDL-C and Apo A-I, and negative correlation of TG, with both eGFR measures. Negative phenotypic correlations of Apo B were observed for both eGFR measures with consistent genotypic correlation for eGFRcys only (Supplementary Figure S3, bottom panel).

**Instrument strength**

The F-statistics for LDL-C, HDL-C and TG showed that the selected instruments (390 variants) were strongly associated with these traits in HUNT (F-statistic: 1814-2475 and R^2^: 2.5-3.4%; Supplementary Table S8B). The conditional F-statistics values were 24.2 for LDL-C, 31.8 for HDL-C and 104.2 for TG. The eGFR-associated GRS explained 1% of the variation in eGFR in the HUNT with a strong F-statistic of 671.5 (Supplementary Table S8B).

Consistently, the selected instruments for these lipid traits in UKBB were strongly associated with the respective traits (F-statistic: 7620-10187 and R^2^: 2.3-4.8%; Supplementary Table S8B). The conditional F-statistics for all 5 lipid traits in UKBB ranged from 703 to 1494 and thus stronger than the values from HUNT. The genetic variants associated with eGFRcrea and eGFRcys explained 3.1% (F-statistic=10314) and 1.5% (F-statistic=4823) variation in these phenotypes, respectively (Supplementary Table S8B).

**GRS associations with confounders**

Among the lipid instruments (Supplementary Figure S4, top panel), the unweighted GRS for LDL-C was weakly associated with age (Supplementary Figure S4A, top panel), and the TG-related GRS was weakly associated with BMI (Supplementary Figure S4C, top panel). Similarly, the GRS for Apo A-1was negatively associated with sex and BMI (Supplementary Figure S4D, top panel), but no such association was observed for the Apo B-related GRS. Moreover, the GRS for eGFRcrea was associated with sex, BMI and current and former smoking (Supplementary Figure S4F, top panel). The GRSs for eGFRcys and UACR were not associated with any of the tested confounders (Supplementary Figure S4G and S4H, top panel).

Similar analyses in UKBB are presented in bottom panel of Supplementary Figure S4. The GRSs for HDL-C, Apo A-I, eGFRcys and UACR were associated with BMI while LDL-C and Apo B GRSs were associated with current smoking. The GRSs for TG and eGFRcrea were not associated with any of the tested confounders (Supplementary Figure S4, bottom panel).

**Reverse multivariable MR**

Multivariable MR in the reverse direction provided some evidence that genetically predicted higher eGFRcys was associated with modestly higher HDL-C and lower TG, with estimates in the opposite direction for eGFRcrea (Supplementary Table S11). The associations of genetically predicted UACR were generally similar in multivariable as in univariable MR, except that multivariable MR also provided evidence that genetically predicted higher UACR was associated with lower HDL-C (-0.11SD; -0.17,-0.04; P=0.001). Sensitivity analysis did not reveal any strong indication of bias due to directional pleiotropy (Supplementary Table S11).

**References**

1. Holmen J, Midthjell K, Krüger Ø, et al. The Nord-Trøndelag Health Study 1995–97 (HUNT 2): objectives, contents, methods and participation. *Norsk epidemiologi* 2003; **13**: 19-32.

2. Study. NTN-TH. [*https://www.ntnu.edu/hunt*](https://www.ntnu.edu/hunt). (accessed on: 18 December 2018) [cited; Available from:

3. Levey AS, Stevens LA, Schmid CH, et al. A new equation to estimate glomerular filtration rate. *Annals of internal medicine* 2009; **150**: 604-12.

4. Friedewald WT, Levy RI, Fredrickson DS. Estimation of the concentration of low-density lipoprotein cholesterol in plasma, without use of the preparative ultracentrifuge. *Clinical chemistry* 1972; **18**: 499-502.

5. Krokstad S, Langhammer A, Hveem K, et al. Cohort profile: the HUNT study, Norway. *International journal of epidemiology* 2012; **42**: 968-77.

6. Das S, Forer L, Schönherr S, et al. Next-generation genotype imputation service and methods. *Nature genetics* 2016; **48**: 1284.

7. McCarthy S, Das S, Kretzschmar W, et al. A reference panel of 64,976 haplotypes for genotype imputation. *Nature genetics* 2016; **48**: 1279.

8. Zhou W, Fritsche LG, Das S, et al. Improving power of association tests using multiple sets of imputed genotypes from distributed reference panels. *Genetic epidemiology* 2017; **41**: 744-55.

9. Zanetti D, Rao A, Gustafsson S, Assimes TL, Montgomery SB, Ingelsson E. Identification of 22 novel loci associated with urinary biomarkers of albumin, sodium, and potassium excretion. *Kidney international* 2019; **95**: 1197-208.

10. Biobank U. Genotyping and quality control of UK Biobank, a large-scale, extensively phenotyped prospective resource. *Available at biobank ctsu ox ac uk/crystal/docs/genotyping_qc pdf Accessed April* 2015; **1**: 2016.

11. Liu DJ, Peloso GM, Yu H, et al. Exome-wide association study of plasma lipids in> 300,000 individuals. *Nature genetics* 2017; **49**: 1758.

12. Richardson TG, Sanderson E, Palmer TM, et al. Evaluating the relationship between circulating lipoprotein lipids and apolipoproteins with risk of coronary heart disease: A multivariable Mendelian randomisation analysis. *PLoS medicine* 2020; **17**: e1003062.

13. Wuttke M, Li Y, Li M, et al. A catalog of genetic loci associated with kidney function from analyses of a million individuals. *Nature genetics* 2019; **51**: 957.

14. Pattaro C, Teumer A, Gorski M, et al. Genetic associations at 53 loci highlight cell types and biological pathways relevant for kidney function. *Nature communications* 2016; **7**: 10023.

15. Teumer A, Li Y, Ghasemi S, et al. Genome-wide association meta-analyses and fine-mapping elucidate pathways influencing albuminuria. *Nature communications* 2019; **10**: 1-19.

16. Zhou W, Nielsen JB, Fritsche LG, et al. Efficiently controlling for case-control imbalance and sample relatedness in large-scale genetic association studies. *Nature genetics* 2018; **50**: 1335.

17. Loh P-R, Tucker G, Bulik-Sullivan BK, et al. Efficient Bayesian mixed-model analysis increases association power in large cohorts. *Nature genetics* 2015; **47**: 284.

18. Yang J, Lee SH, Goddard ME, Visscher PM. GCTA: a tool for genome-wide complex trait analysis. *The American Journal of Human Genetics* 2011; **88**: 76-82.

19. Lee SH, Yang J, Goddard ME, Visscher PM, Wray NR. Estimation of pleiotropy between complex diseases using single-nucleotide polymorphism-derived genomic relationships and restricted maximum likelihood. *Bioinformatics* 2012; **28**: 2540-2.

20. Zheng J, Erzurumluoglu AM, Elsworth BL, et al. LD Hub: a centralized database and web interface to perform LD score regression that maximizes the potential of summary level GWAS data for SNP heritability and genetic correlation analysis. *Bioinformatics* 2017; **33**: 272-9.

21. Bulik-Sullivan B, Finucane HK, Anttila V, et al. An atlas of genetic correlations across human diseases and traits. *Nature genetics* 2015; **47**: 1236.

22. Hemani G, Bowden J, Davey Smith G. Evaluating the potential role of pleiotropy in Mendelian randomization studies. *Human molecular genetics* 2018; **27**: R195-R208.

23. Choi SW, Mak TSH, O'reilly P. A guide to performing Polygenic Risk Score analyses. *BioRxiv* 2018: 416545.

24. Sanderson E, Windmeijer F. A weak instrument F-test in linear IV models with multiple endogenous variables. *Journal of Econometrics* 2016; **190**: 212-21.

25. Sanderson E, Smith GD, Windmeijer F, Bowden J. An examination of multivariable Mendelian randomization in the single sample and two-sample summary data settings. *bioRxiv* 2018: 306209.

26. Bowden J, Smith GD, Haycock PC, Burgess S. Consistent estimation in Mendelian randomization with some invalid instruments using a weighted median estimator. *Genetic epidemiology* 2016; **40**: 304-14.

27. Bowden J, Davey Smith G, Burgess S. Mendelian randomization with invalid instruments: effect estimation and bias detection through Egger regression. *International journal of epidemiology* 2015; **44**: 512-25.

28. Rees JM, Wood AM, Burgess S. Extending the MR‐Egger method for multivariable Mendelian randomization to correct for both measured and unmeasured pleiotropy. *Statistics in medicine* 2017; **36**: 4705-18.

29. Hemani G, Tilling K, Smith GD. Orienting the causal relationship between imprecisely measured traits using GWAS summary data. *PLoS genetics* 2017; **13**: e1007081.

30. Hemani G, Zheng J, Elsworth B, et al. The MR-Base platform supports systematic causal inference across the human phenome. *Elife* 2018; **7**: e34408.
